# Genetic Risk Meets the Intestinal Barrier

**DOI:** 10.1111/imcb.70158

**Published:** 2026-08-14

**Authors:** J. L. E. Tearle, K. R. James

**Affiliations:** ^1^ Translational Genomics Program Garvan Institute of Medical Research Darlinghurst, Sydney New South Wales Australia; ^2^ School of Biomedical Sciences University of New South Wales Sydney New South Wales Australia

## Abstract

Top: At increasing cellular resolution, the Alegbe et al. report that expression quantitative trait loci (eQTLs) are enriched in enhancer regions rather than promoters. They speculate that enhancer eQTLs only come into effect during certain cellular contexts, making them more tolerable. Bottom: Alegbe et al. identify an enrichment of eQTLs affecting Wnt pathway regulators in inflammatory bowel disease (IBD) epithelium, suggesting a reduced capacity for regeneration. Additionally, the authors show an enrichment of interaction eQTLs (ieQTLs) in IBD epithelium that become active only during inflammation.

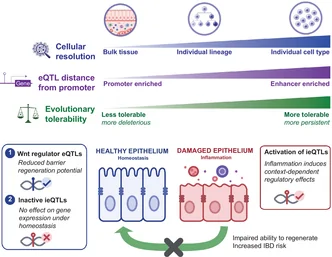

Inflammatory bowel disease (IBD) arises from the breakdown in intestinal barrier function and immune homeostasis leading to chronic inflammation. Currently, 180 000 Australians live with IBD and the incidence is rising. Genetic factors are a known driver of IBD susceptibility. In fact, the leading risk factor for IBDs, including Crohn's disease (CD) and ulcerative colitis (UC), is a blood relative, that is, a sibling, parent, or child, already diagnosed, and twin studies show a 50% concordance in CD incidence in monozygotic twins versus 10% in dizygotic twins. Making sense of how genetic variants drive IBD holds significance in disease prevention and treatment choice.

Genome‐Wide Association Studies (GWAS) have linked over 300 genetic modifications with conventional polygenic IBD. Among associated genes are *Nucleotide‐binding oligomerization domain containing 2* (*NOD2*), *IL23R*, and *ATG16L1*, with roles in immune regulation, microbial recognition, and autophagy—pointing to potential pathogenic pathways. However, the vast majority of IBD‐associated loci have unknown roles in cell responses or disease pathways, principally because 90% of GWAS genes map to noncoding regions. These noncoding variations may instead drive changes in the regulation of gene expression to promote IBD onset.

Large‐scale expression quantitative trait loci (eQTL) studies have aimed to unite noncoding GWAS variants with their gene of effect with some success. In particular, these studies have focused on cis‐eQTLs, variants regulating expression of a nearby gene on the same chromosome. However, most GWAS loci remain without an effector gene. Barriers to the success of eQTL studies are that most studies are conducted with bulk sequencing, which averages gene expression across diverse cell types and masks potentially cell type‐specific gene regulation. Additionally, studies with peripheral blood mononuclear cells may not recapitulate genetic programs at play in disease‐relevant tissue. Emerging single‐cell RNA sequencing studies have demonstrated eQTLs operating at the cell type and state levels, but few have characterized this in the context of disease, including IBD. This frontier is a critical next step in unraveling IBD disease biology and dysregulation of gene expression in disease more generally.

In a landmark study [1], Alegbe and colleagues leverage large‐scale single‐cell eQTL mapping across peripheral blood *and* disease‐relevant intestinal tissue to construct the most comprehensive map of genetic regulation in IBD to date. The authors build the IBDverse (also published in a complementary paper [2]), an extensive single‐cell dataset with over 1.8 million cells from terminal ileum and peripheral blood of over 400 individuals including 125 with IBD. They annotated this dataset with 86 transcriptionally distinct cell groups across nine major cell lineages. In examining genetic effects across these cell annotations, the authors uncover previously hidden dimensions of disease biology, revealing not only where genetic risk acts but also how it translates to intestinal dysfunction.

The authors first asked: What happens when we look at genetic regulation at different cell resolutions? They performed eQTL mapping at three levels: bulk (all cells), broad cell lineages, and at individual cell group resolution. While bulk data harbored the largest number of eGenes (genes with at least one significant eQTL), the regulatory landscape shifted markedly at higher resolution, with cell‐type‐specific eQTLs occurring progressively further from their target genes and becoming increasingly enriched within enhancer regions rather than promoters. Notably, disease‐associated variants identified by GWAS are similarly enriched within enhancer regions, further suggesting that cellular resolution is essential for capturing the regulatory effects most relevant to disease (Figure 1, top).

**FIGURE 1:**
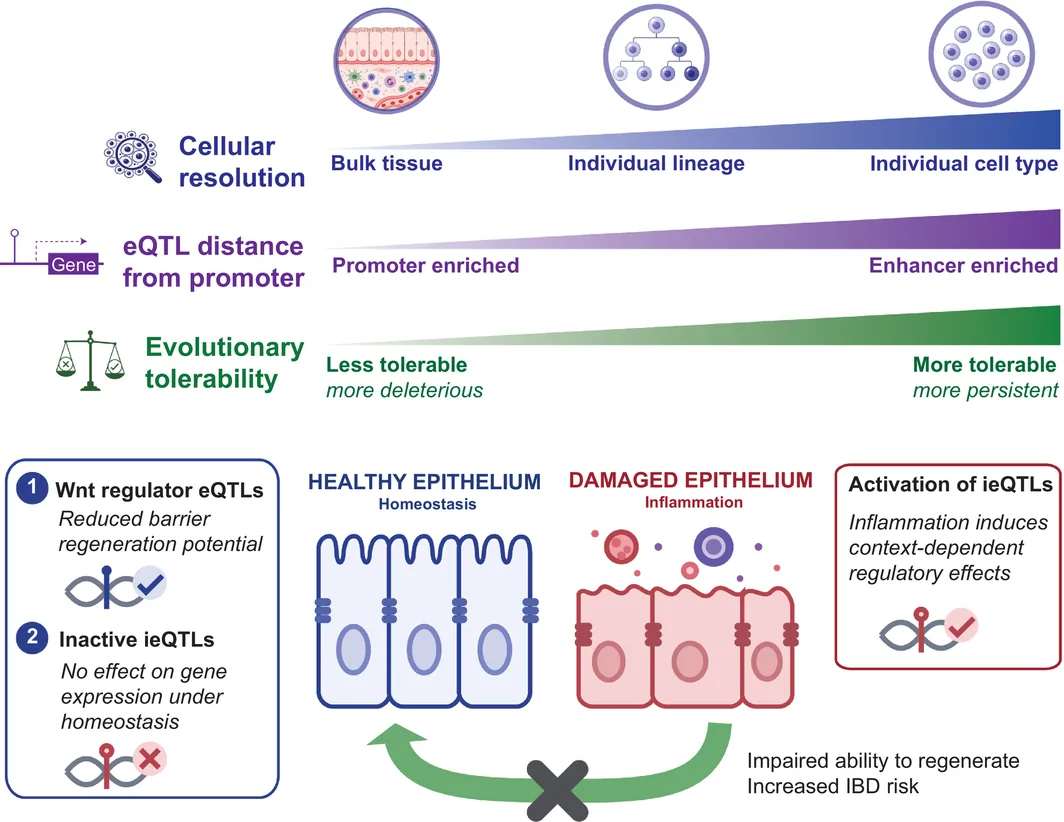
Top: At increasing cellular resolution, the Alegbe et al. report that expression quantitative trait loci (eQTLs) are enriched in enhancer regions rather than promoters. They speculate that enhancer eQTLs only come into effect during certain cellular contexts, making them more tolerable. Bottom: Alegbe et al. identify an enrichment of eQTLs affecting Wnt pathway regulators in inflammatory bowel disease (IBD) epithelium, suggesting a reduced capacity for regeneration. Additionally, the authors show an enrichment of interaction eQTLs (ieQTLs) in IBD epithelium that become active only during inflammation.

But why should disease‐associated variants preferentially reside within enhancer elements? One explanation put forward by Alegbe et al. is that unlike promoter variants, which can influence gene expression broadly across different cell types and states, enhancer variants often exert effects only in the presence of cell‐type‐specific transcription factors. Such context‐dependent regulation may be more evolutionarily tolerable than broadly acting promoter variants, allowing risk alleles to remain hidden until the appropriate biological context arises. In fact, the authors demonstrate this concept, identifying more than 1000 inflammation‐dependent eQTLs (interaction eQTLs, or ieQTLs), raising the possibility that genetic susceptibility and inflammatory activity reinforce one another during disease progression.

When it comes to novel IBD biology revealed by Alegbe and colleagues' approach, the epithelium takes center stage. Strikingly, the majority of inflammation‐dependent regulatory effects were observed in enterocytes, highlighting the epithelium as a major site where genetic susceptibility and inflammatory cues converge.

The true power of this study becomes apparent when the authors integrate their disease‐relevant tissue eQTL maps with known IBD‐associated genetic variants derived from GWAS, addressing the long‐standing gap between genetic association and biological mechanism. While disease‐associated variants were linked to multiple cellular compartments, a consistent theme emerged within epithelial cells, where several independent risk loci converged on epithelial pathways involved in barrier maintenance and regeneration. Most notably, the authors mapped a total of 20 risk loci to Wnt regulators, of which only six had been previously reported (Figure 1, bottom). Additionally, the canonical Wnt target *MYC* was linked to a CD risk variant in intestinal stem cells, collectively suggesting that impaired tissue maintenance and repair are fundamental components of disease risk rather than simply downstream consequences of inflammation.

Beyond biological discovery, the framework described by Alegbe and colleagues has important implications for therapeutic development. The authors successfully recovered several established IBD drug targets within their colocalization analyses, including *JAK2* (tofacitinib) and *ITGA4* (vedolizumab), while also identifying new candidate targets and providing human genetic support for emerging therapies such as PRKCB inhibition. Importantly, their analyses also suggest a role for disease‐relevant eQTL maps in anticipating on‐target toxicities, exemplified by *PSEN2*, where genetic evidence links perturbation of Notch signaling to impaired epithelial homeostasis.

More broadly, the epithelial biology emerging from this study raises important questions about how IBD should be treated. Current therapies overwhelmingly target immune pathways, yet the genetic architecture revealed here suggests that impaired epithelial maintenance and repair may be a fundamental component of disease susceptibility. These findings also have implications for understanding host–microbiome interactions in IBD; if genetic susceptibility impairs epithelial renewal and barrier resilience, microbial and dietary exposures may exert a disproportionate influence on disease development, not necessarily because they are intrinsically pathogenic, but because they are acting upon a tissue less capable of maintaining homeostasis following injury. As human genetics increasingly implicates pathways governing tissue resilience and regeneration, future therapeutic strategies may need to move beyond suppressing inflammation alone and toward restoring the capacity of the intestinal mucosa to repair itself following injury.

By bringing human genetics into the diseased intestine, Alegbe and colleagues reveal biology that has so far remained hidden by averaging across tissues and studying circulating immune cells in isolation. Ultimately, they remind us: if we want to understand complex disease, we must study it in the place and context where it unfolds.

## Author Contributions

**J. L. E. Tearle:** conceptualization, writing – original draft, writing – review and editing. **K. R. James:** conceptualization, writing – original draft, writing – review and editing.

## Conflicts of Interest

The authors declare no conflicts of interest.

## Data Availability

The authors have nothing to report.
